# Self‐Determined Motivation and Physical Activity in Chronic Obstructive Pulmonary Disease

**DOI:** 10.1111/crj.70209

**Published:** 2026-06-30

**Authors:** Larissa Guimarães Paiva, Nara Batista de Souza, Vitória Abraão de Lima, Vitória Lourdes de Oliveira Lima, Manuela Karloh, Jhon Alefe Abreu dos Santos, Cristino Carneiro Oliveira, Klaus Chaves Alberto, Daniela Pereira Almeida, Anderson José, Carla Malaguti

**Affiliations:** ^1^ Graduate Program in Health, Faculty of Medicine Federal University of Juiz de Fora Juiz de Fora Minas Gerais Brazil; ^2^ Physical Therapy College Federal University of Juiz de Fora Juiz de Fora Minas Gerais Brazil; ^3^ Center for Assistance, Education, and Research in Pulmonary Rehabilitation, Behavior Change, and Hospital Physical Therapy ‐ NuReab. Center of Health and Sport Sciences – CEFID Universidade do Estado de Santa Catarina – UDESC Florianópolis Santa Catarina Brazil; ^4^ Physical Therapy Graduate Program ‐ PPGFT. Center of Health and Sport Sciences – CEFID Universidade do Estado de Santa Catarina – UDESC Florianópolis Santa Catarina Brazil; ^5^ Graduate Program in Rehabilitation Sciences Federal University of Minas Gerais – UFMG, and Federal University of Espírito Santo – UFES Vitória Espírito Santo Brazil; ^6^ Graduate Program in Built Environment Federal University of Juiz de Fora Juiz de Fora Minas Gerais Brazil; ^7^ Graduate Program in Architecture and Urbanism Federal University of Viçosa Viçosa Minas Gerais Brazil; ^8^ Graduate Program in Rehabilitation Sciences and Physical‐Functional Performance Federal University of Juiz de Fora Juiz de Fora Minas Gerais Brazil

**Keywords:** accelerometry, COPD, motivation, physical activity, self‐determination theory

## Abstract

**Background:**

Chronic Obstructive Pulmonary Disease (COPD) is associated with reduced physical activity, negatively affecting prognosis and quality of life. According to Self‐Determination Theory (SDT), the quality of motivation plays a key role in sustaining active behavior, but evidence in COPD remains limited.

**Objective:**

The objective of this study was to examine the associations between different types of exercise motivation and objectively measured physical activity and sedentary behavior in individuals with COPD.

**Methods:**

This cross‐sectional study included 50 community‐dwelling individuals with COPD (GOLD stages 2–3; mean age 69.4 ± 7 years). Motivation was assessed using the Behavioral Regulation in Exercise Questionnaire (BREQ‐3) and the Self‐Determination Index (SDI). Physical activity was objectively measured with the ActiGraph GT3X accelerometer. Associations were examined using Spearman's correlations and hierarchical multiple linear regression models.

**Results:**

Integrated regulation was positively correlated with light physical activity (rho = 0.43, *p* < 0.05), total physical activity (rho = 0.40, *p* < 0.05), and daily step count (rho = 0.43, *p* < 0.05). Intrinsic motivation was positively correlated with light physical activity (rho = 0.37, *p* < 0.05), total physical activity (rho = 0.37, *p* < 0.05), and daily step count (rho = 0.35, p < 0.05). The Self‐Determination Index was positively associated with light physical activity (rho = 0.28, p < 0.05) and daily step count (rho = 0.33, p < 0.05), but showed no significant association with sedentary time. In hierarchical regression analyses, higher levels of integrated regulation were independently associated with higher daily step count, with each one‐unit increase associated with 687 additional steps per day.

For total physical activity, integrated regulation showed a borderline independent association with the outcome after adjustment for clinical covariates (β = 0.279, ***p*** = 0.054), whereas FEV_1_ (% predicted) remained independently associated with total physical activity (β = 0.304, ***p*** = 0.027). For sedentary behavior, higher self‐determination was independently associated with lower sedentary time; specifically, each one‐unit increase in the Self‐Determination Index corresponded to approximately 13 fewer sedentary minutes per day (β = −0.327, *p* = 0.026).

**Conclusion:**

Self‐determined motivation was associated with more favorable physical activity profiles in individuals with COPD. Integrated regulation was independently associated with daily step count, while higher self‐determination was associated with lower sedentary time. These findings suggest that autonomous forms of motivation may be important factors to consider when promoting active lifestyles in people with COPD.

## Graphical abstract

In individuals with COPD, higher autonomous motivation is associated with greater physical activity and lower sedentary behavior measured by accelerometry. Integrated regulation emerged as the main motivational driver of daily activity and step count. Targeting motivational processes may help support more active lifestyles in COPD.

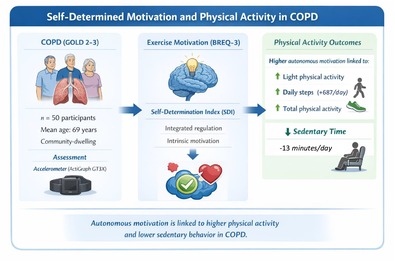

## 1 Introduction

Chronic Obstructive Pulmonary Disease (COPD) is a progressive lung condition that leads to airflow limitation, respiratory symptoms, and systemic consequences, resulting in reduced exercise tolerance and quality of life [1]. Physical inactivity and sedentary behavior are well‐documented predictors of morbidity and mortality in COPD patients [2, 3, 4], so promoting regular physical activity is essential for slowing disease progression, improving prognosis, and enhancing daily functioning [5]. However, behavioral and psychological factors, particularly motivation, may strongly influence patients' engagement in physical activity [6, 7, 8].

According to Self‐Determination Theory (SDT), the quality of motivation that drives human behavior varies along a continuum reflecting the degree of self‐determination. At the autonomous end of this continuum, regulation stems from volition, personal choice, and the internalization of values. Autonomous motivation includes intrinsic motivation, characterized by engaging in an activity for its inherent satisfaction or interest; and two types forms of extrinsic motivation: integrated regulation, in which the behavior is fully aligned with one's sense of self and core values; and identified regulation, where the person consciously values the behavior and accepts it as personally important. In contrast, controlled motivation arises when behavior is regulated by internal or external pressures. It encompasses introjected regulation, driven by feelings of guilt, obligation, or self‐esteem contingencies, and external regulation, in which actions are performed to obtain rewards or avoid punishment [6, 9, 10].

Within this framework, autonomous types of motivation, particularly integrated regulation, have been associated with greater persistence and engagement in physical activity, as exercise is more fully integrated into individuals' values, lifestyle, and sense of self. Motivation in health contexts can be assessed using instruments grounded in SDT, such as the Treatment Self‐Regulation Questionnaire (TSRQ) [11], the Exercise Self‐Regulation Questionnaire (SRQ‐E) [12], and the Behavioral Regulation in Exercise Questionnaire (BREQ) series [13]. Among these, the BREQ‐3 is a comprehensive tool, encompassing all six motivational regulations proposed by SDT: amotivation, external, introjected, identified, integrated, and intrinsic regulations. Previous studies in chronic conditions suggest that autonomous motivation, and especially integrated regulation, are positively associated with adherence to physical activity [14, 15]; however, little is known about how these motivational patterns relate to objectively measured physical activity in individuals with COPD.

This study aimed to investigate the associations between different types of exercise motivation regulations and two behavioral outcomes in patients with COPD, physical activity and sedentary behavior. We hypothesized that higher autonomous motivation would be associated with greater physical activity and less sedentary behavior in this population.

## 2 Materials and Methods

### 2.1 Study Design and Participants

This cross‐sectional study was reported according to STROBE recommendations, reports a secondary analysis of baseline data from the research project “Exploring the impact of the environment on physical activity in patients with chronic obstructive pulmonary disease (EPCOT)‐A comparative analysis between suggested and free walking: Protocol study [16]”, conducted at the Federal University of Juiz de Fora, Minas Gerais, Brazil. Data collection was conducted between September 2023 and October 2024, following approval by the institutional ethics committee (protocol #5.972.823). All participants signed an informed consent. Participants were recruited from primary healthcare units within the municipality through telephone or in‐person contact. The study has been completed and is no longer recruiting participants.

For the present analysis, only baseline data were used, when no intervention was applied. A total of 50 patients with COPD were included. Eligibility criteria were: diagnosis of COPD according to GOLD criteria (post‐bronchodilator FEV_1_/FVC < 70%), clinically stable condition, ability to complete assessments, and not involved in rehabilitation programs in the last 6 months. Exclusion criteria included other severe cardiorespiratory, neurological, or musculoskeletal disorders, recent hospitalization (< 3 months), or use of long‐term oxygen therapy.

### 2.2 Assessments

#### 2.2.1 Pulmonary Function

Pulmonary function was performed using a calibrated spirometer (Datospir Micro C, Sibelmed, Spain, and Spirobank II Advanced, Italy) following the American Thoracic Society/European Respiratory Society technical statement [17]. Reference equations were used for predicted values [18]. The severity of airway obstruction was classified according to the GOLD criteria [1].

#### 2.2.2 Symptom Burden

The COPD Assessment Test (CAT) was used to assess the impact of COPD. This questionnaire measures the impact of COPD on a person's life and how it changes over time. Each question is scored from 0 (not impaired) to 5 points (maximally impaired). The total score ranged from 0 to 40, with higher scores reflecting a greater burden of the disease [19].

#### 2.2.3 Breathlessness

Perception of breathlessness in daily living was evaluated using the Modified Medical Research (MMRC) dyspnea scale [20], which consists of five statements that describe almost the entire range of dyspnea from none (Grade 0) to almost complete incapacity (Grade 4).

#### 2.2.4 Exercise Motivation

Exercise motivation was assessed using the Behavioral Regulation in Exercise Questionnaire (BREQ‐3) [12], a validated instrument in Portuguese [21] for assessing motivational regulations and self‐determination. The BREQ‐3 assesses different types of motivational regulations along the self‐determination continuum. The scale includes: amotivation (lack of intention to exercise), external regulation (exercise driven by external rewards or pressures), introjected regulation (exercise motivated by internal pressures such as guilt or obligation), identified regulation (recognition of the personal value of exercise), integrated regulation (exercise aligned with personal values and identity), and intrinsic regulation (exercise performed for inherent enjoyment and satisfaction). Each item is rated on a 5‐point Likert scale ranging from 0 (“not true for me”) to 4 (“very true for me”), with higher scores indicating stronger endorsement of the corresponding type of motivational regulation.

For analysis, scores can be interpreted both at the individual regulation level and as a composite measure using the Self‐Determination Index (SDI), which weights autonomous and controlled forms of motivation to provide an overall indicator of self‐determined motivation. Higher SDI values reflect greater autonomous motivation, associated with more consistent engagement in physical activity, while lower or negative values indicate predominance of controlled motivation or amotivation [12]. In this study, the BREQ‐3 motivational regulations and the SDI were used as key behavioral outcomes to explore associations with objectively measured physical activity and sedentary behavior.

#### 2.2.5 Physical Activity

Physical activity was assessed objectively using the ActiGraph GT3X, a triaxial accelerometer worn at the iliac crest level of the dominant lower limb that captures free‐living activity [22]. The accelerometer was configured to collect data at 85.7 Hz. For inclusion in the analysis, participants were required to have at least 4 valid days of wear, including at least one weekend day [23, 24]. A valid day was defined as having at least 10 h of wear time within 24 h, and only waking periods were retained for analysis [25], Raw accelerometer data were processed using ActiLife software, and activity counts were converted to METs (1 MET = 3.5 mL/kg/min) using the Freedson Adult equation implemented in ActiLife software. Physical activity intensity was classified according to absolute‐intensity thresholds as sedentary (< 1.5 METs), light (1.5–2.99 METs), moderate (3.00–5.99 METs), and vigorous (> 6 METs). These thresholds are widely used in accelerometer‐based research and facilitate comparisons with current physical activity recommendations and previous studies in COPD populations [25]. Daily total physical activity was calculated as the sum of time spent in light, moderate, and vigorous‐intensity physical activity. To account for differences in the number of valid monitoring days between participants, all physical activity and sedentary behavior variables were normalized and expressed as average minutes per day by dividing the accumulated values by the number of valid wear days. Sedentary time was analyzed separately and was not included in the total physical activity variable. Sedentary behavior was derived from the vertical axis counts using established cut‐points, with periods ≤ 100 counts per minute classified as sedentary [26, 27]. Non‐wear time was identified as ≥ 60 consecutive minutes of zero counts, allowing a 2‐min interruption. Step counts were extracted from the ActiGraph's built‐in algorithm, providing an objective measure of ambulatory activity. This approach allowed for a comprehensive assessment of both overall physical activity and sedentary behavior in free‐living conditions.

### 2.3 Statistical Analysis

Analyses were performed using the Statistical Package for the Social Sciences Version 24 (IBM Corp., Armonk, New York). As this was a cross‐sectional study based on available data, a post hoc power analysis for the bivariate correlation (two‐tailed, α = 0.05, effect size r = 0.4, *n* = 50) indicated a statistical power of 83%.

Descriptive statistics were expressed as mean ± standard deviation (SD) for normally distributed variables and as median and interquartile range (IQR) for non‐normally distributed variables. Associations between motivational variables and physical activity and sedentary behavior outcomes were analyzed using Spearman's rank correlation coefficients, given the non‐normal distribution of motivational data. Correlation strength was interpreted as follows: values < 0.30 indicated weak correlations, 0.30–0.50 moderate correlations, and > 0.50 strong correlations [28].

Hierarchical multiple linear regression analyses were performed to examine the associations between motivational constructs and physical activity and sedentary behavior outcomes. Based on theoretical and empirical evidence, clinically relevant variables previously reported to be associated with physical activity in COPD (age, sex, forced expiratory volume in 1 s [FEV_1_], modified Medical Research Council dyspnea scale [mMRC], and COPD Assessment Test [CAT]) were entered in the first block. Motivational variables were subsequently entered in the second block to assess their additional contribution beyond established clinical determinants. This approach was chosen to account for potential confounding factors and to evaluate the incremental explanatory value of motivational constructs. Results are presented as standardized regression coefficients (β), 95% confidence intervals (95% CI), the coefficient of determination (R^2^), and the change in explained variance (ΔR^2^). When more than one motivational construct showed a significant bivariate association with a given outcome, only the construct with the strongest association and greatest theoretical relevance was entered into the regression model to avoid multicollinearity and improve model stability. A *p*‐value < 0.05 was considered statistically significant.

## 3 Results

### 3.1 Participant Flow and Characteristics

**FIGURE 1:**
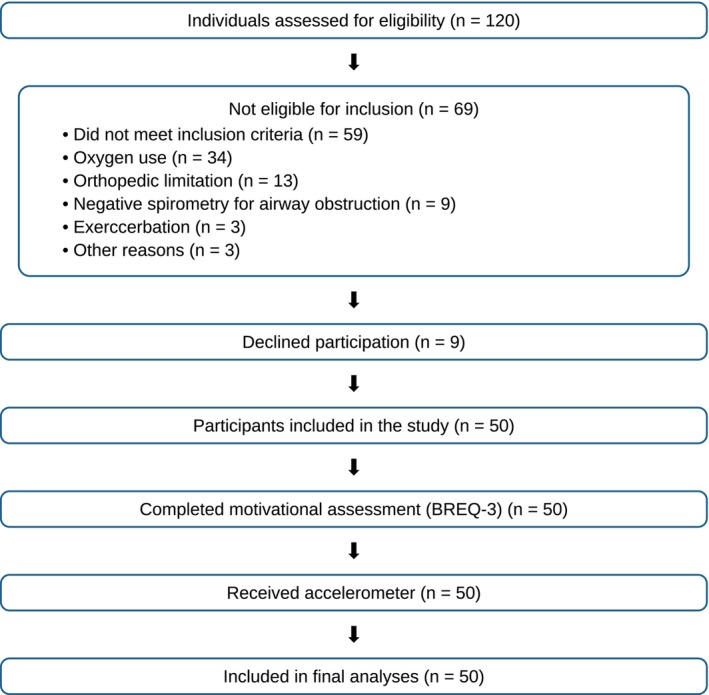
Participant flow diagram showing the recruitment process and inclusion in the final analyses.

A total of 120 individuals were assessed for eligibility. Sixty‐one individuals were not eligible for inclusion, primarily due to oxygen use, orthopedic limitations, absence of airflow obstruction on spirometry, recent exacerbation, or other reasons, and nine declined participation. Consequently, 50 participants were included in the study (Figure 1). All included participants completed the study assessments and were included in the analyses. The participant flow is presented in Figure 1. Participants' mean age was 69.4 ± 7 years, 54% were female, most (84%) had GOLD Stages 2–3 COPD, and mean FEV_1_ was 57.3% ± 19.5% predicted (Table 1).

**TABLE 1:** Participant characteristics (*n* = 50).

Variable	Values
Age (years)	69.4 ± 7.0
Female sex	27 (54)
mMRC dyspnea	1.8 ± 1.0
Symptom Burden, CAT score	17.2 ± 7.7
BMI	26.5 ± 6.0
FEV_1_, % pred	57.3 ± 19.5
GOLD Stages 2–3	42 (84)
Sedentary time (min/day)	685.53 ± 330.4
LIPA (min/day)	285.3 ± 135.0
MPA (min/day)	9.2 ± 11.9
VPA (min/day)	0.2 ± 0.6
Daily total physical activity (min)	297.6 ± 135.2
Step number	4196.2 ± 2203.5
Amotivation	0.0 (0.0–1.0)
External regulation	1.0 (0.0–2.6)
Introjected regulation	1.3 (0.0–2.7)
Identified regulation	3.0 (2.7–4.0)
Integrated regulation	2.4 (1.4–3.8)
Intrinsic motivation	2.9 (1.5–4.0)
Self‐determination index	10.8 (1.3–17.4)

*Note:* Results are means and standard deviation, median and interquartile range, or absolute and relative values.

Abbreviations: BMI: body mass index; BREQ‐3: Behavioral Regulation Exercise Questionnaire; CAT: COPD assessment test; FEV_1_: forced expiratory volume in 1 s; FVC: forced vital capacity; GOLD: Global Initiative for Chronic Obstructive Lung Disease; LIPA: light intensity physical activity; mMRC: modified medical research council; MPA: moderate intensity physical activity; VPA: vigorous intensity physical activity.

### 3.2 Correlation Analysis

Higher levels of autonomous motivation were associated with more favorable physical activity profiles (Table 2). Integrated regulation showed the strongest associations, being positively correlated with light physical activity (r = 0.43, *p* < 0.05), total physical activity (r = 0.40, *p* < 0.05), and daily step count (r = 0.43, *p* < 0.05), while being inversely associated with vigorous physical activity (r = −0.29, *p* < 0.05). Intrinsic motivation exhibited a similar pattern, showing positive correlations with light physical activity (r = 0.37, *p* < 0.05), total physical activity (r = 0.37, *p* < 0.05), and daily step count (r = 0.35, *p* < 0.05). Introjected regulation was positively associated with light physical activity (r = 0.31, *p* < 0.05) and total physical activity (r = 0.30, *p* < 0.05), whereas being inversely associated with vigorous physical activity (r = −0.30, *p* < 0.05). Higher Self‐Determination Index scores were positively correlated with light physical activity and daily step count, whereas no significant correlation was observed with sedentary time. Amotivation was negatively correlated with vigorous physical activity (r = −0.29, *p* < 0.05).

**TABLE 2:** Associations between motivational variables and physical activity and sedentary behavior outcome variables.

Motivation type	Sedentary time	Light PA	Moderate PA	Vigorous PA	Total PA	Steps number
Amotivation	0.18	0.21	−0.17	**−0.29** *	0.12	0.05
External						
External regulation	0.10	−0.02	−0.45	−0.11	0.02	−0.01
Introjected regulation	−0.05	**0.31** *	−0.20	**−0.30** *	**0.30** *	0.09
Identified regulation	−0.27	0.17	0.01	−0.15	0.16	0.16
Integrated regulation	−0.21	**0.43** *	0.26	**−0.29** *	**0.40** *	**0.43** *
Intrinsic						
Intrinsic motivation	−0.15	**0.37** *	0.15	−0.26	**0.37** *	**0.35** *
Total						
Self‐Determination index	−0.24	**0.28** *	0.22	−0.11	0.26	**0.33** *

Abbreviation: PA = physical activity. Bold values indicate statistical significance (*p* < 0.05).

* *p* < 0.05.

### 3.3 Regression Analysis

Hierarchical multiple linear regression analyses were conducted to examine the associations between motivational constructs and physical activity outcomes after accounting for clinically relevant variables. For daily step count, the clinical model (Model 1) identified dyspnea as the only variable independently associated with the outcome. After the inclusion of integrated regulation (BREQ) in Model 2, integrated regulation was independently associated with daily step count, whereas the association between dyspnea and step count was no longer statistically significant. These findings suggest that higher levels of integrated regulation are associated with a greater number of daily steps, independent of age, sex, dyspnea, and lung function (Tables 3 and 4).

**TABLE 3:** Hierarchical multiple linear regression analysis examining associations between integrated regulation and daily step count.

Variables	Model 1				Model 2			
	B	β	95% CI	*p*	B	β	95% CI	*p*
Constant	9204.047	—	2688.475–15719.619	0.007	5307.241	—	−1135.938–11750.419	0.104
Age	−65.355	−0.204	−149.24–22.51	0.144	−53.155	−0.178	−133.89–23.08	0.162
Sex	544.819	0.127	−645.76–1760.75	0.356	381.804	0.085	−730.61–1476.65	0.499
FEV_1_ (% predicted)	15.840	0.135	−16.07–46.45	0.333	24.428	0.221	−4.29–54.03	0.093
CAT	−16.582	−0.058	−125.233	0.760	17.185	0.060	−84.233–118.604	0.734
mMRC	−743.959	−0.383	−1433.01 to −228.13	**0.008**	−516.435	−0.201	−1039.41–167.47	0.152
Integrated regulation	—	—	—	—	687.459	0.427	247.04–1096.21	**0.003**

*Note:* Sex was coded as 0 = female and 1 = male. Model 1 included age, sex, FEV_1_ (% predicted), CAT score and mMRC score. Model 2 additionally included integrated regulation (BREQ—3).

Abbreviations: B = unstandardized regression coefficient; CAT = COPD Assessment Test; FEV_1_ = forced expiratory volume in 1 s; mMRC = modified Medical Research Council Dyspnea Scale; β = standardized regression coefficient; 95% CI = 95% confidence interval. Bold values indicate statistical significance (*p* < 0.05).

**TABLE 4:** Hierarchical multiple linear regression model summary for daily step count.

Model	R^2^	Adjusted R^2^	ΔR^2^	F	*p*
Model 1	0.216	0.147	0.216	3.107	0.024
Model 2	0.363	0.291	0.147	10.163	0.003

*Note:* R^2^ = coefficient of determination; Adjusted R^2^ = adjusted coefficient of determination; ΔR^2^ = change in explained variance between models. Model 1 included age, sex, FEV_1_ (% predicted), CAT and mMRC score. Model 2 additionally included integrated regulation (BREQ 3). F change and *p* values indicate the statistical significance of the increase in explained variance after inclusion of integrated regulation.

For total physical activity, the clinical model (Model 1) did not identify any independent correlates of the outcome. After the inclusion of integrated regulation (Model 2), FEV_1_ (% predicted) was independently associated with total physical activity, whereas integrated regulation showed a borderline association (*p* = 0.054). These findings suggest that better lung function is associated with higher levels of total physical activity, while integrated regulation may contribute to physical activity behavior beyond clinical characteristics (Tables 5 and 6).

**TABLE 5:** Hierarchical multiple linear regression analysis for total physical activity.

Variables	Model 1				Model 2			
	B	β	95% CI	p	B	β	95% CI	p
Constant	2229.666	—	646.701–3812.632	0.007	1599.202	—	−64.033–3262.436	0.059
Age	−15.099	−0.192	−36.426–6.229	0.161	−13.125	−0.167	−33.890–7.641	0.209
Sex	97.291	0.088	−198.900–393.483	0.511	70.917	0.064	−217.375–359.209	0.622
FEV_1_ (% predicted)	7.267	0.255	−0.479–15.013	0.065	8.657	0.304	1.018–16.296	**0.027**
CAT	−11.599	−0.160	−37.995–14.798	0.381	−6.135	−0.084	−32.316–20.045	0.639
mMRC	−155.044	−0.283	−357.282–47.194	0.129	−118.233	−0.216	−317.780–81.314	0.239
Integrated regulation	—	—	—	—	111.224	0.279	−2.163–224.611	0.054

*Note:* Sex was coded as 0 = female and 1 = male. Model 1 included age, sex, FEV_1_ (% predicted), CAT score and mMRC score. Model 2 additionally included integrated regulation (BREQ—3).

Abbreviations: B = unstandardized regression coefficient; CAT = COPD Assessment Test; FEV_1_ = forced expiratory volume in 1 s; β = standardized regression coefficient; 95% CI = 95% confidence interval; mMRC = modified Medical Research Council Dyspnea Scale. Bold values indicate statistical significance (*p* < 0,05).

**TABLE 6:** Model summary of hierarchical regression for total physical activity.

Model	R^2^	Adjusted R^2^	ΔR^2^	F	*p*
Model 1	0.280	0.198	0.280	3.425	0.011
Model 2	0.340	0.248	0.060	3.695	0.005

*Note:* R^2^ = coefficient of determination; Adjusted R^2^ = adjusted coefficient of determination; ΔR^2^ = change in explained variance between models. Model 1 included age, sex, FEV_1_ (% predicted), CAT and mMRC score. Model 2 additionally included integrated regulation (BREQ‐3). F change and *p* values indicate the statistical significance of the increase in explained variance after inclusion of integrated regulation.

For sedentary behavior, the clinical model (Model 1) identified mMRC as the only variable independently associated with sedentary time. After the inclusion of the Self‐Determination Index (SDI) in Model 2, SDI was independently and inversely associated with sedentary time, whereas the association between mMRC and sedentary behavior was no longer statistically significant. These findings suggest that higher levels of self‐determination are associated with lower sedentary time, independent of demographic and clinical characteristics (Tables 7 and 8).

**TABLE 7:** Hierarchical multiple linear regression analysis for sedentary behavior.

Variables	Model 1				Model 2			
	B	β	95% CI	*p*	B	β	95% CI	*p*
Constant	625.205	—	−365.242–1615.651	0.210	867.472	—	−101.361–1836.305	0.078
Age	1.711	0.037	−11.633–15.055	0.797	1.089	0.023	−11.661–13.839	0.864
Sex	114.275	0.174	−71.049–299.599	0.221	131.980	0.201	−45.608–309.568	0.141
FEV_1_ (% predicted)	−3.157	−0.187	−8.003–1.690	0.196	−2.928	−0.173	−7.559–1.703	0.209
CAT	−10.333	−0.240	−26.849–6.183	0.214	−13.580	−0.316	−29.600–2.439	0.095
mMRC	135.220	0.416	8.682–261.759	**0.037**	114.217	0.352	−7.961–236.395	0.066
Self‐Determination Index	—	—	—	—	−13.195	−0.327	−24.705 to −1.686	**0.026**

*Note:* Sex was coded as 0 = female and 1 = male. Model 1 included age, sex, FEV_1_ (% predicted), CAT score and mMRC score. Model 2 additionally included Self‐Determination Index (BREQ‐3).

Abbreviations: B = unstandardized regression coefficient; FEV_1_ = forced expiratory volume in 1 s; β = standardized regression coefficient; 95% CI = 95% confidence interval; mMRC = modified Medical Research Council Dyspnea Scale. Bold values indicate statistical significance (*p* < 0.05).

**TABLE 8:** Model summary of hierarchical regression for sedentary behavior.

Model	R^2^	Adjusted R^2^	ΔR^2^	F	*p*
Model 1	0.169	0.105	0.169	2.149	0.07
Model 2	0.285	0.185	0.089	2.858	0.02

*Note:* R^2^ = coefficient of determination; Adjusted R^2^ = adjusted coefficient of determination; ΔR^2^ = change in explained variance between models. Model 1 included age, sex, FEV_1_ (% predicted), CAT and mMRC score. Model 2 additionally included Self‐Determination Index (BREQ‐3). F change and *p* values indicate the statistical significance of the increase in explained variance after inclusion of Self‐Determination Index.

## 4 Discussion

This study demonstrated that more autonomous motivation was associated with higher levels of physical activity and lower levels of sedentary behavior. These findings support the central premise of Self‐Determination Theory, namely that the quality of motivation, rather than its quantity, is associated with engagement in physical activity. More self‐determined types of regulation, such as integrated, are positively associated with consistent participation in physical activity and inversely related to sedentary time. Among individuals with COPD, this motivational pattern is particularly salient: physical limitations such as dyspnea and fatigue may undermine perceptions of autonomy and competence, thereby constraining the internalization of physical activity behaviors. Overall, the results support the hypothesis that higher levels of autonomous motivation correspond to greater physical activity and lower sedentary behavior in this population, in line with SDT tenets.

An unexpected finding was the inverse correlation between integrated regulation and vigorous physical activity. This result should be interpreted with caution given the extremely low levels of vigorous activity observed in the sample (0.2 ± 0.6 min/day). The highly skewed distribution of vigorous physical activity, with many participants likely accumulating little or no vigorous activity, may have contributed to unstable correlation estimates. Furthermore, vigorous activity is generally uncommon among individuals with COPD, and greater integrated regulation may be more strongly reflected in increases in light‐ and moderate‐intensity activities, daily step count, and overall physical activity rather than engagement in vigorous‐intensity exercise. This interpretation is supported by the positive associations observed between integrated regulation, total physical activity, and daily step count.

The small‐to‐moderate effect of motivation observed (β = 0.279 for total physical activity and β = 0.427 for step count) highlights its relevance even in the presence of disease‐related limitations. Previous evidence indicates that physical activity in COPD is influenced by multiple physiological and clinical factors, including exercise capacity, muscle dysfunction, cardiovascular comorbidities, and systemic manifestations of the disease [29, 30, 31, 32, 33, 34]. Our findings suggest that motivational processes may represent an additional and potentially modifiable determinant of physical activity behavior. By addressing both physiological and psychological factors, interventions may more effectively reduce inactivity and improve long‐term health outcomes in COPD. Furthermore, pulmonary rehabilitation programs have been encouraged to incorporate strategies that support self‐determined forms of exercise motivation, consistent with the principles of Self‐Determination Theory [35, 36].

Although motivation was associated with higher levels of physical activity, its relationship with sedentary behavior also deserves consideration. Although sedentary behavior is often attributed to physiological impairment and symptom burden [37], our findings also indicate that motivational factors may represent an additional and potentially modifiable determinant of sedentary time. The moderate independent association between SDI and sedentary behavior suggests that interventions targeting autonomous motivation could complement traditional rehabilitation approaches focused primarily on symptom management and exercise capacity.

From a clinical perspective, higher levels of integrated regulation were associated with approximately 687 additional daily steps and 111 additional minutes of total physical activity; however, the association narrowly missed statistical significance (*p* = 0.054), whereas higher self‐determination was associated with approximately 13 fewer sedentary minutes per day. These findings support the relevance of motivation as a factor associated with daily movement behavior in individuals with COPD and suggest that it may represent a promising target for behavioral interventions. Indeed, evidence from isotemporal substitution and epidemiological studies suggests that reallocating short periods of sedentary time to light or moderate physical activity can yield measurable health benefits [38].

Our findings align with previous research showing that autonomous motivation is associated with exercise behavior in COPD [39]. However, unlike previous studies, we did not observe significant associations between controlled regulations and physical activity. This discrepancy may reflect differences in study context. Whereas prior investigations were conducted within structured rehabilitation programs, the present study examined free‐living behavior, where daily activity choices may depend more strongly on internalized motives than on external contingencies [38, 40]. These findings further support the role of autonomous motivation as a key determinant of physical activity engagement in COPD.

Together with our findings, this evidence reinforces the importance of fostering autonomous rather than controlled motivation. According to SDT, supporting the basic psychological needs for autonomy, competence, and relatedness may facilitate long‐term engagement in physical activity [29, 40, 41, 42, 43, 44]. Accordingly, interventions should promote enjoyment, volitional choice, personal meaning, and social support as key mechanisms for sustaining exercise behavior [7, 41].

A key strength of this study is the use of objective measures of physical activity and sedentary behavior, which provide more reliable estimates compared to self‐reported data. Moreover, the application of the BREQ‐3, which captures all six types of motivational regulation for exercise, expands previous research limited to older versions of the instrument or rehabilitation‐based samples. In addition, participants were community‐dwelling individuals with COPD who had never participated in pulmonary rehabilitation programs, which may enhance the ecological validity of the findings by reflecting real‐world behavior patterns and contributes to understanding motivation in COPD. A notable finding of this study is that it extends current knowledge by examining associations between motivational regulation, particularly autonomous forms, and both physical activity and sedentary behavior in individuals with COPD. Beyond establishing theoretical associations, our findings provide clinically relevant evidence that motivation should not be viewed solely as a contextual factor, but rather as a measurable and potentially modifiable target in rehabilitation. This expands the conventional scope of pulmonary rehabilitation by suggesting that promoting autonomous motivation, rather than relying on external incentives, education, or compliance‐driven strategies, can meaningfully be associated to daily movement behavior. These results offer an important message for rehabilitation professionals: fostering autonomy, competence, and relatedness may be as vital as prescribing exercise itself, positioning motivation at the core of long‐term engagement and behavioral sustainability.

This study has some limitations. The cross‐sectional design precludes causal inferences, and the single‐center sample may limit the generalizability of the results. In addition, the study was conducted at a single center and included predominantly clinically stable individuals with moderate COPD (mainly GOLD Stages 2–3); therefore, the findings may not be generalized to patients with more severe disease or those experiencing acute clinical instability. Although motivation was assessed by self‐report, the use of a comprehensive and validated instrument (BREQ‐3) provides greater confidence in the observed associations. The relatively small sample size may have limited the precision and stability of the regression estimates, despite the use of parsimonious models including a limited number of clinically relevant covariates. The number of statistical comparisons performed may have increased the risk of Type I error. Although the analyses were guided by a priori hypotheses and the main findings were further examined in adjusted hierarchical regression models, the results should be interpreted with caution and confirmed in larger studies. Future longitudinal studies are warranted to confirm these relationships and to investigate the prospective associations between different types of motivation and changes in physical activity and sedentary behavior over time in individuals with COPD.

In conclusion, this study highlights the association between autonomous forms of motivation, higher levels of physical activity, and lower sedentary behavior in individuals with COPD. Although causality cannot be inferred, these findings suggest that autonomous motivation may play an important role in supporting more active lifestyles in this population. Integrating motivational approaches and evidence‐based behavior change techniques into exercise prescription, guided by established frameworks such as SDT, may enhance engagement in pulmonary rehabilitation and support the long‐term adoption and maintenance of physically active lifestyles beyond structured programs.

## Author Contributions

LGP: Conceptualization; Investigation; Data curation; Formal analysis; Visualization; Writing – original draft; NBS: Investigation; Data curation; Writing – review and editing; VAL: Investigation; Data curation; Writing – review and editing; VLOL: Investigation; Data curation; Writing – review and editing; MK: Methodology; Validation; Writing – review and editing; JAAS: Software; Formal analysis; Visualization; Writing – review and editing; CCO: Methodology; Validation; Writing – review and editing; KCA: Resources; Project administration; Writing – review and editing; DPA: Supervision; Methodology; Writing – review and editing; AJ: Supervision; Funding acquisition; Project administration; Writing – review and editing; CM: Conceptualization; Methodology; Supervision; Funding acquisition; Project administration; Validation; Writing – review and editing.

## Funding

This study was supported by Conselho Nacional de Desenvolvimento Científico e Tecnológico (CNPq; grant number 401882/2023‐3) and Coordenação de Aperfeiçoamento de Pessoal de Nível Superior (CAPES; Finance Code 001). The funding agencies had no role in the study design, data collection, analysis, interpretation of data, manuscript preparation, or decision to publish.

## Ethics Statement

This study was approved by the Institutional Review Board (protocol number 5.972.823) and conducted in accordance with the Declaration of Helsinki.

## Conflicts of Interest

The authors declare no conflicts of interest.

## Data Availability

The data that support the findings of this study are available on request from the corresponding author. The data are not publicly available due to privacy or ethical restrictions.
